# Drug-induced urinary retention: a real-world pharmacovigilance study using FDA and Canada vigilance databases

**DOI:** 10.3389/fphar.2024.1466875

**Published:** 2025-01-06

**Authors:** Xianyu Dai, Kai Yu, Yu Chang, Yuchuan Hou

**Affiliations:** ^1^ Department of Urology, The First Hospital of Jilin University, Changchun, China; ^2^ Department of Gastroenterology, The First Hospital of Jilin University, Changchun, China

**Keywords:** urinary retention, adverse events, FAERS, Canadian Vigilance Adverse Reaction (CVAR), pharmacovigilance

## Abstract

**Background:**

Urinary retention (UR) is a clinical condition where patients cannot fully empty their bladder. Although numerous drugs are associated with UR, comprehensive and reliable studies identifying drugs that induce UR are scarce.

**Methods:**

This study leveraged data from the FDA Adverse Event Reporting System (FAERS) and the Canadian Vigilance Adverse Reaction (CVAR) database to explore adverse events (AEs) related to UR from 2004 to Q1 2024. The top 50 drugs were analyzed for annual reporting trends using linear regression. Disproportionality analysis using the reporting odds ratio (ROR) method, with *P*-values adjusted via Bonferroni correction, identified significant signals, which were then validated against drug labels and re-evaluated using the CVAR database. Time-to-onset analysis was also performed.

**Results:**

From 2004 to Q1 2024, FAERS recorded 17,785,793 AEs, with 16,183 (0.09%) identified as UR cases. The median age among these cases was 65 years, with males comprising 53.4%. There were significant annual increases in UR reports associated with antineoplastic agents (0.19% per year) and antidiabetic drugs (0.09% per year), while reports linked to bronchodilators decreased (−0.53% per year). Disproportionality analysis revealed significant signals for 34 drugs (68%), with the highest RORs observed in Fesoterodine, Mirabegron, and Solifenacin. Initial signal detection identified potential new UR signals for Abiraterone, Valacyclovir, Fluoxetine, Empagliflozin, Clopidogrel, and Amlodipine, with CVAR confirming signals for Abiraterone, Fluoxetine, and Empagliflozin. The median time to onset of UR was 29 days, with over half of the cases occurring within 30 days of initiating medication.

**Conclusion:**

The study identifies a rising trend in drug-related UR reports over the past 2 decades. The validation of new signals for Abiraterone, Fluoxetine, and Empagliflozin underscores the critical need for continuous drug safety monitoring and targeted research to better understand the mechanisms behind drug-induced UR.

## 1 Introduction

Urinary retention (UR) is a common yet serious clinical condition characterized by the inability of patients to completely empty their bladder. UR can be classified into acute and chronic types. Acute UR typically presents as sudden onset difficulty in urination and a sensation of bladder fullness, often requiring urgent medical interventions such as catheterization or surgical treatment (Thomas et al., 2004). Chronic UR may lead to bladder overdistension, recurrent urinary tract infections, and bladder stone formation, significantly reducing the patient’s quality of life (Pape and Nitti, 2018).

The pathogenesis of UR is complex and may involve multiple factors such as bladder outlet obstruction, neurological dysfunction, urinary tract inflammation, or adverse drug effect (Selius and Subedi, 2008). Bladder outlet obstruction may be caused by conditions like benign prostatic hyperplasia or urethral stricture. Neurological dysfunctions that impair normal bladder contraction include diseases such as multiple sclerosis, Parkinson’s disease, stroke, and spinal cord injuries (Moussa et al., 2020). Observational studies indicate that up to 10% of UR cases may be attributable to medication use (Verhamme et al., 2008). Previous reports have identified drugs associated with UR, including methamphetamine, sertraline, and buprenorphine (Edwards et al., 2014; Ojo et al., 2021; Jiang et al., 2022).

Crisafulli et al. previously utilized the Italian Spontaneous Reporting System database, which primarily collects and records reports from Italy, to explore drugs that might induce UR (Crisafulli et al., 2022). However, this study involved only 421 reports of adverse events (AEs) related to UR and lacked further validation from external databases, limiting its comprehensiveness and accuracy.

The aim of this study is to utilize two large databases, FDA Adverse Event Reporting System (FAERS, the world’s largest post-marketing safety surveillance database) and Canadian Vigilance Adverse Reaction database (CVAR), to systematically and comprehensively investigate and analyze adverse drug reaction events related to UR. We also analyzed the onset time of AEs associated with drug-induced UR to further understand the temporal patterns of drug-induced UR. This study aims to provide a scientific basis for drug safety surveillance and to offer clinical references to improve medication safety for patients.

## 2 Materials and methods

### 2.1 Study design and data source

This study initially utilizes the FAERS database to extract reports of AEs related to UR. The top 50 drugs most commonly associated with UR were identified and categorized for annual reporting trend analysis. Disproportionality analysis was performed on these 50 drugs to explore UR-related AE signals, which were then matched against the drug labels to identify any discrepancies. For drugs without labeled UR, further validation was conducted using the CVAR database. FAERS contains millions of real-world AEs reports submitted by healthcare professionals, individual patients, lawyers, and drug manufacturers. The FAERS data files include seven types of datasets: demographic and administrative information (DEMO), drug information (DRUG), adverse event coding (REAC), patient outcomes (OUTC), report sources (RPSR), therapy start and end dates (THER), and indications for drug use (INDI) (Kadoyama et al., 2012). Each report categorizes the role of each drug in the AE: primary suspect (PS), secondary suspect (SS), interacting (I), or concomitant (C).

To ensure the reliability of results, we extracted UR-related AEs reports submitted by healthcare professionals (including physicians, pharmacists, and other health professionals) between 2004 and the first quarter of 2024. Considering the various sources of FDA data submissions, potential duplicate reports were handled following FDA guidelines: when CASEID was the same, we selected the latest FDA_DT and the highest PRIMARYID. The CVAR database, managed by Health Canada, has recorded post-market adverse reactions in Canada since 1965, including patient characteristics, drug usage, adverse reactions, and outcomes.

### 2.2 Identification of target AE reports

All reported AEs were coded in detail according to the Medical Dictionary for Regulatory Activities (MedDRA) classification system. MedDRA’s hierarchical structure includes five levels: System Organ Class (SOC), High-Level Group Term (HLGT), High-Level Term (HLT), Preferred Term (PT), and Lowest Level Term (LLT) (Mascolo et al., 2021). In this study, we extracted all AE reports containing the PT “urinary retention” and primarily focused on drugs listed as “PS”.

### 2.3 Statistical analysis

This study evaluated annual reporting trends of the top 50 drug-related categories using time series plots and linear regression analysis, with *P*-values adjusted using the Bonferroni method. Disproportionality analysis, a common method in pharmacovigilance, based on the classical 2 × 2 contingency table (Table 1), was used to analyze the frequency of target drug and target AE occurrences relative to background frequencies, establishing statistical associations between drugs and AEs. The reporting odds ratio (ROR) algorithm was employed to detect drug-related AE signals. The ROR and its 95% confidence interval (CI) were calculated as follows:

$$R\text{OR}=\frac{ad}{bc},95\%\text{ CI}=e^{\ln\left(ROR\right)\;\pm 1.96\sqrt{\left(\frac{1}{a}+\frac{1}{b}+\frac{1}{c}+\frac{1}{d}\right)}}$$

**TABLE 1:** 2x2 contingency table for disproportionality analysis.

	Target AEs	All other AEs
Target drug	a	b
All other drugs	c	d

Notes: “a” represents the number of specific AEs, related to the target drug combination, “b” represents the number of other AEs related to the target drug, “c” represents the number of AEs related to other drugs involving the target AE, and “d” represents the number of other AEs unrelated to the target drug. AEs, adverse events.

A positive AEs signal was identified when the lower limit of the 95% CI for the ROR was greater than 1.0, with at least 3 reports of the target AE (a ≥3) and P-adjust < 0.05. P-adjust is the *p*-value adjusted by chi-square test and Bonferroni correction. The ROR value also serves as an indicator to compare AEs risks among drugs; a higher ROR value suggests a higher risk of drug-induced UR (Yang et al., 2022). All analyses were conducted using R software version 4.2.3.

### 2.4 Time-to-onset analysis

The time-to-onset was defined as the interval from the therapy start date (START_DT in the THER file) to the event date (EVENT_DT in the DEMO file). Reports with input errors (e.g., EVENT_DT earlier than START_DT), inaccurate dates, and duplicates were excluded. In this study, the median and quartiles were used to describe the time-to-onset of AEs.

## 3 Results

### 3.1 Baseline characteristics of UR

**FIGURE 1:**
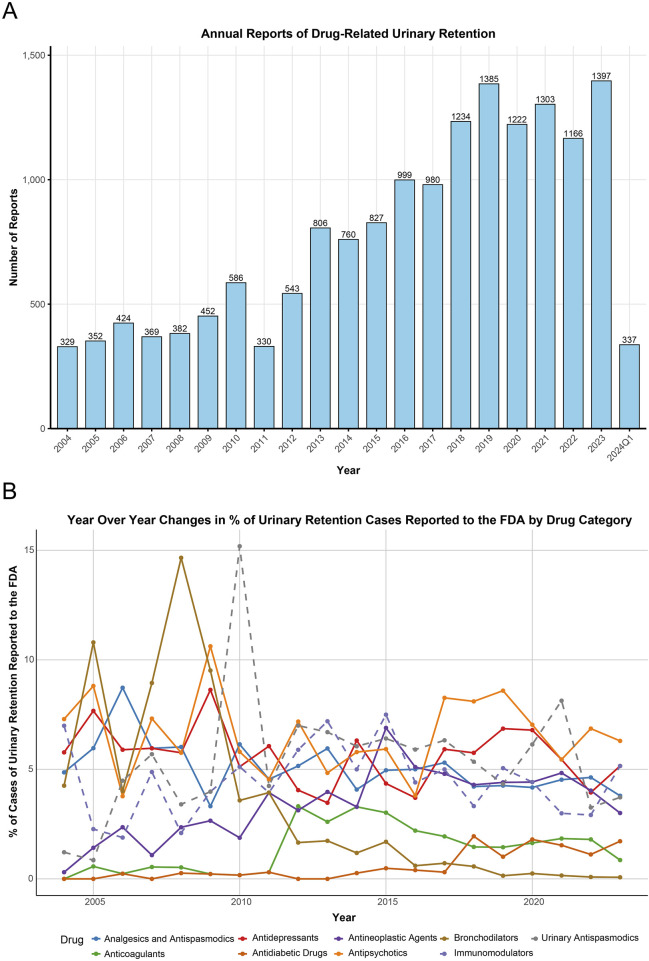
Annual reporting trends and time series plot. **(A)** The annual trend in the number of adverse event reports related to urinary retention from 2004 to the first quarter of 2024. **(B)** Changes in the percentage of urinary retention cases reported to the FAERS associated with various drug classes from 2004 to 2023. FAERS, FDA Adverse Event Reporting System.

During the study period from 2004 to Q1 2024, the FDA reported a total of 17,785,793 AEs, of which 16,183 (0.09%) were UR cases reported by healthcare professionals. Table 2 describes the baseline characteristics of patients with drug-related UR. Overall, reports of drug-related UR showed an increasing trend (Table 2; Figure 1A), with the highest number of reports in 2023 (1,397 cases, 8.63%). Among patients experiencing drug-related UR, males (53.4%) were more prevalent than females (37.2%), with a median age of 65 years (interquartile range [IQR] 47.0, 76.0) and a median weight of 72 kg (IQR 59.0, 86.2). Physicians accounted for the largest proportion of reports (51.9%), followed by other healthcare workers (35.9%). The United States reported the highest number of cases (33.0%), followed by Japan (10.6%), France (8.8%), the United Kingdom (8.3%), and Germany (6.0%). Details of case reports from other countries can be found in Supplementary Table S1.

**TABLE 2:** Basic characteristics of patients with drug-related UR from the FAERS database. UR, Urinary retention.

Characteristics	Drug-related UR (N = 16,183)
Gender	
Male	8,641 (53.4%)
Female	6,027 (37.2%)
Unknown	1,515 (9.4%)
Age (years)	
Median (Q1, Q3)	65.0 (47.0, 76.0)
Unknown	4,332 (26.8%)
Weight (kg)	
Median (Q1, Q3)	72.0 (59.0, 86.2)
Unknown	11,107 (68.6%)
Reported person	
Physician	8,399 (51.9%)
Pharmacist	1975 (12.2%)
Other health-professional	5,809 (35.9%)
Reported countries	
United States	5,348 (33.0%)
Japan	1712 (10.6%)
France	1,431 (8.8%)
United Kingdom	1,344 (8.3%)
Germany	976 (6.0%)
Canada	756 (4.7%)
Others^a^	4,616 (28.5%)
Reporting year	
2004–2008	1856 (11.5%)
2009–2013	2,717 (16.8%)
2014–2018	4,800 (29.7%)
2019–2023	6,473 (40.0%)
2024 Q1	337 (2.0%)

Notes:

^a^ See Supplementary Table S1 for other countries.

### 3.2 Trend analysis of drug-related UR incidents

This study analyzed the top 50 drugs related to UR reported to the FDA (Supplementary Table S2). The drug categories included Immunomodulators (8/50), Antidepressants (7/50), Antipsychotics (6/50), Antineoplastic Agents (6/50), Analgesics and Antispasmodics (5/50), Urinary Antispasmodics (5/50), Anticoagulants (3/50), Antidiabetic Drugs (2/50), Bronchodilators (1/50), and Others (7/50). The time series of these drug reports is shown in Figure 1B. Additionally, linear regression analyses were conducted for each major drug category related to UR (Table 3). Regression for Antineoplastic Agents showed an average annual increase of 0.19% (95% CI: 0.10, 0.28, p-adjust = 0.004) from 0.30% in 2004 to 3% in 2023, a faster growth rate than any other drug category. Regression analysis for Antidiabetic Drugs also showed an average annual increase of 0.09% (95% CI: 0.06, 0.12, p-adjust < 0.001) in UR reports to the FDA. Conversely, the proportion of UR reports related to Bronchodilators showed a declining trend (−0.53% per year, 95% CI: −0.75, −0.31, p-adjust < 0.001). Other drug categories (Analgesics and Antispasmodics, Anticoagulants, Antidepressants, Antipsychotics, Immunomodulators, Urinary Antispasmodics) showed stable trends over time (*p*-adjust > 0.05).

**TABLE 3:** Linear regression analysis of the percentage of urinary retention cases associated with different drug classes. For each drug class, the slope, 95% CI, P-adjust, and the percentages in 2004 and 2023 are included. CI, confidence interval.

Reports of urinary retention to FDA from 2004 to 2023 by drug category					
Drug category	% change per year (95% CI)	% in 2004	% in 2023	*p*-value	p-adjust^a^
Analgesics and Antispasmodics	−0.11 (−0.19, −0.03)	4.86	3.79	0.012	0.105
Anticoagulants	0.08 (0.01, 0.16)	0	0.86	0.031	0.277
Antidepressants	−0.07 (−0.16, 0.03)	5.78	5.15	0.206	1.000
Antipsychotics	−0.01 (−0.15, 0.13)	7.29	6.30	0.905	1.000
Antineoplastic Agents	0.19 (0.10, 0.28)	0.30	3.00	<0.001	**0.004**
Antidiabetic Drugs	0.09 (0.06, 0.12)	0	1.72	<0.001	**<0.001**
Immunomodulators	0.01 (−0.11, 0.14)	6.99	5.15	0.843	1.000
Urinary Antispasmodics	0.09 (−0.14, 0.31)	1.22	3.72	0.463	1.000
Bronchodilators	−0.53 (−0.75, −0.31)	4.26	0.07	<0.001	**0.001**

Notes: ^a^ *P*-values were adjusted using the Bonferroni method. Bold values indicate statistically significant p-adjust.

### 3.3 Signal detection and validation

**FIGURE 2:**
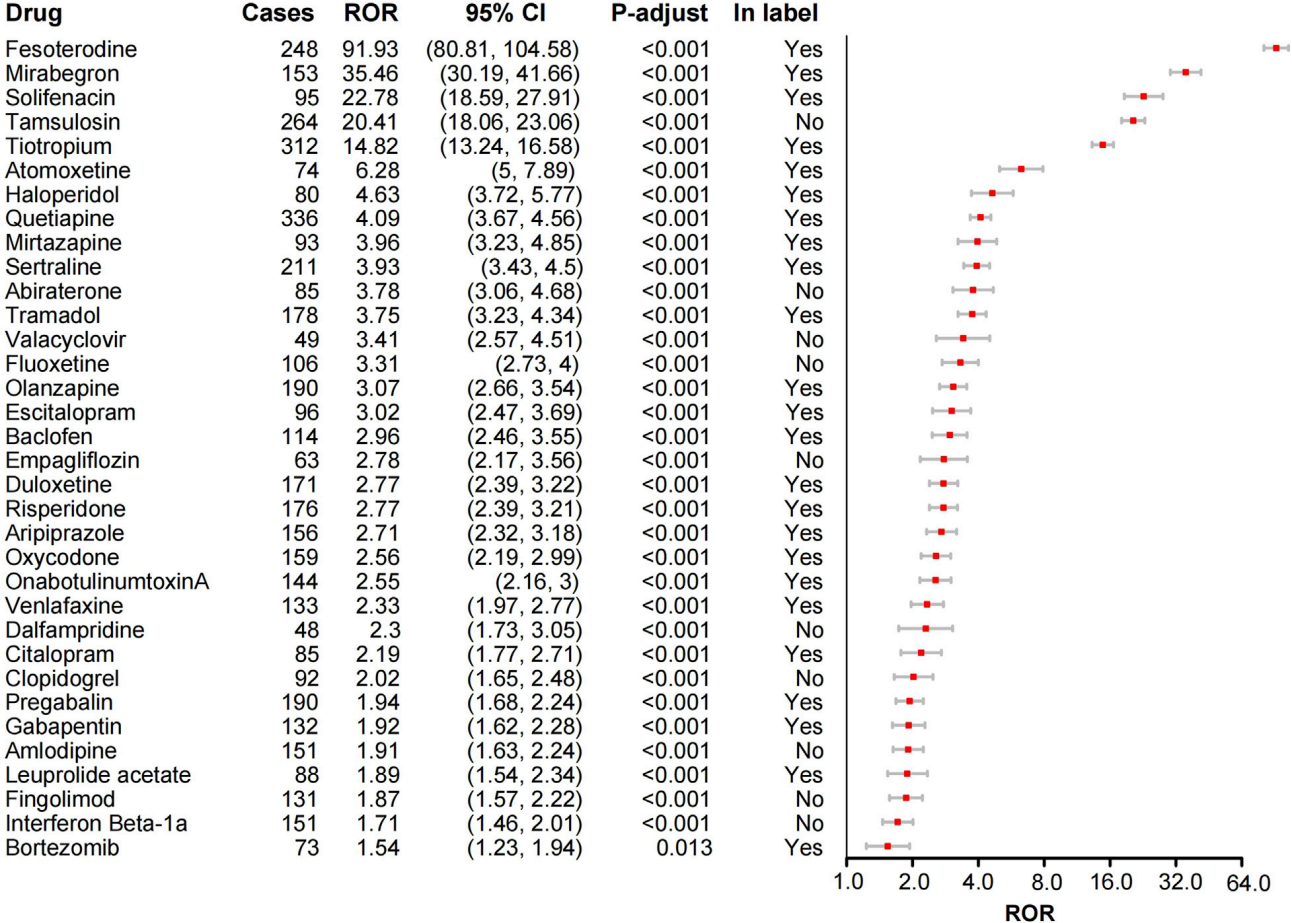
Forest plot of ROR analysis for 34 drugs with positive urinary retention signals and label information. ROR, Reporting odds ratio. CI, confidence interval. *P*-values were adjusted using the Bonferroni method.

**FIGURE 3:**
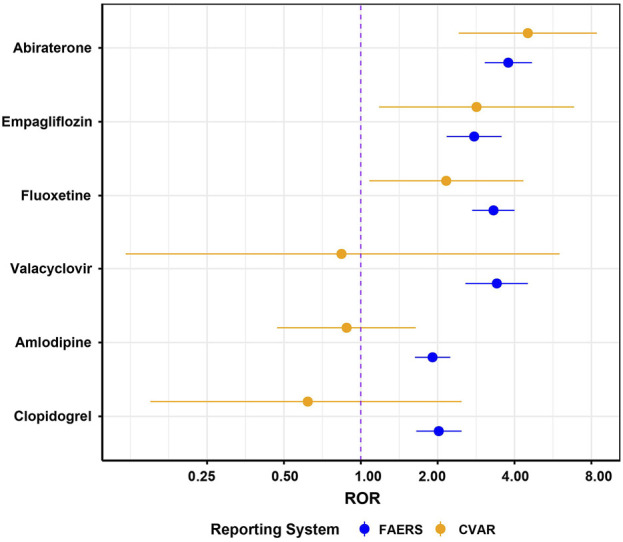
Analysis of ROR for drug adverse reactions related to urinary retention not covered in drug instructions, based on FAERS and CVAR. ROR, Reporting odds ratio. FAERS, FDA Adverse Event Reporting System. CVAR, Canada Vigilance Adverse Reaction Online Database.

The ROR method was applied to the top 50 drugs for AE signal detection (Supplementary Table S2). The drugs with the most UR reports were Quetiapine (n = 336), followed by Tiotropium (n = 312), Tamsulosin (n = 264), Fesoterodine (n = 248), and Lenalidomide (n = 246). After Bonferroni correction, 34 drugs (68%) exhibited significant signals for UR (Figure 2). The top five drugs by signal strength were Fesoterodine (ROR = 91.93), Mirabegron (ROR = 35.46), Solifenacin (ROR = 22.78), Tamsulosin (ROR = 20.41), and Tiotropium (ROR = 14.82). Notably, some drugs such as Fesoterodine, Mirtazapine, and Sertraline explicitly mentioned UR as a potential adverse reaction on their labels, consistent with our findings (Figure 2). Additionally, we discovered some drugs not listed for UR in their labels (Figure 2). However, certain drugs, like Tamsulosin for benign prostatic hyperplasia and Dalfampridine, Fingolimod, and Interferon Beta-1a for multiple sclerosis, are not considered new findings as their indications inherently risk UR. After screening, Abiraterone, Valacyclovir, Fluoxetine, Empagliflozin, Clopidogrel, and Amlodipine were identified as drugs with unexpected UR potential. Subsequently, we validated these unexpected findings using the CVAR database with the ROR method. Positive signals for Abiraterone, Fluoxetine, and Empagliflozin were confirmed (Figure 3), indicating a high risk of inducing UR.

### 3.4 Onset time of UR

**FIGURE 4:**
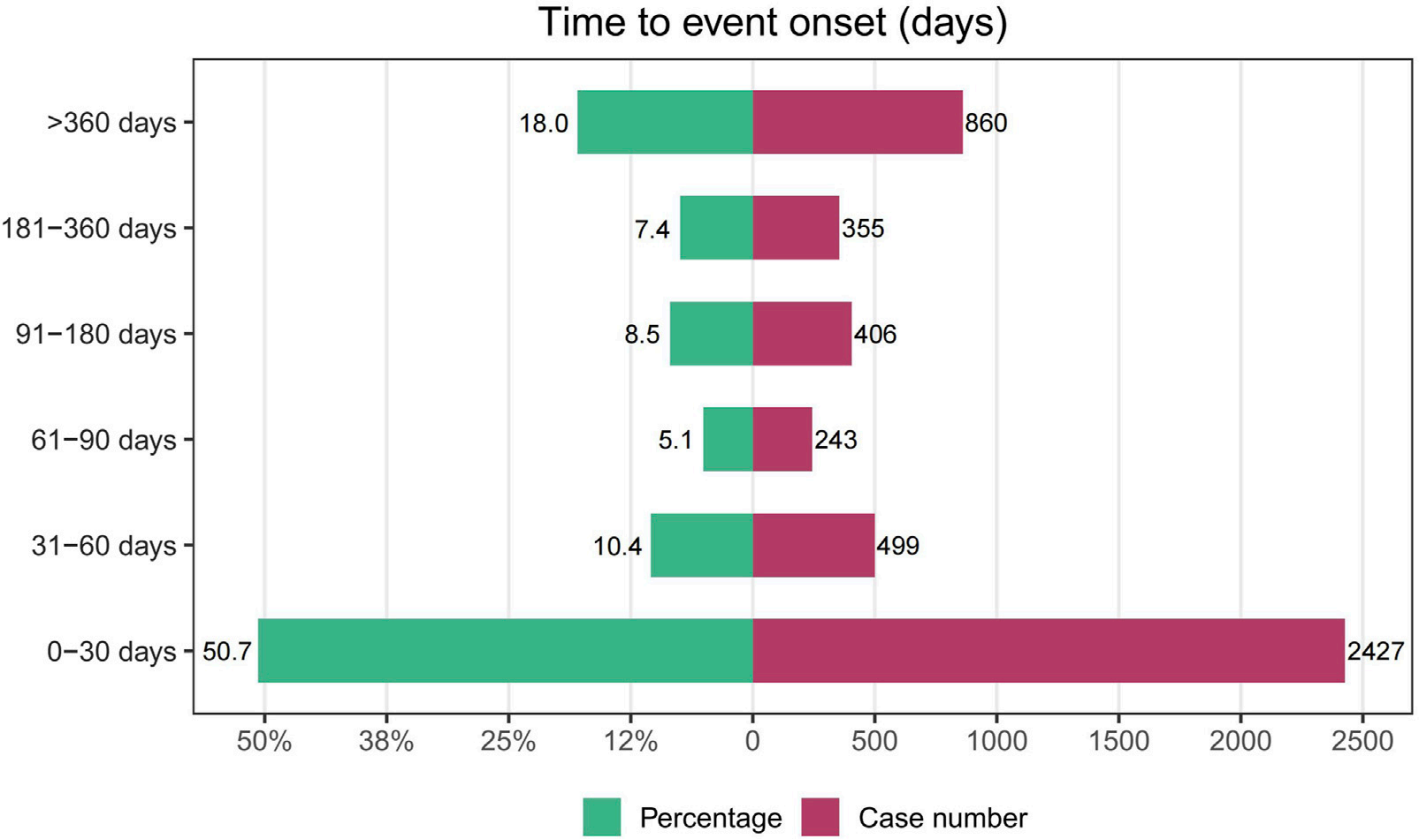
Time to onset of drug-related urinary retention.

After removing duplicates and erroneous reports, 4,790 reports provided onset time data. The median time to onset for drug-related UR was 29 days (IQR 6–183 days). Most cases of UR occurred within 30 days of medication initiation (n = 2,427, 50.7%), but UR could still occur over a year after starting the medication (n = 860, 18%), as shown in Figure 4.

## 4 Discussion

To our knowledge, this is the first study to jointly utilize the FAERS and CVAR databases to mine and analyze AEs related to drug-induced UR. Compared to previous studies based solely on the Italian spontaneous reporting system database (Crisafulli et al., 2022), our study features a larger sample size of AE reports (N = 16,183) and includes only data reported by healthcare professionals. Our findings were rigorously adjusted using the Bonferroni correction. This study reveals reporting trends of common drug categories associated with UR-related AEs from 2004 to 2023, using statistical methods to quantify these trends—an analysis that previous studies lacked. Further signal detection identified six drugs related to UR that were not mentioned on the product labels. Of these, three drugs—Abiraterone, Fluoxetine, and Empagliflozin—were further validated using the CVAR database, enhancing the reliability of our results.

During the past 2 decades, we observed a significant upward trend in reports of drug-related UR (Table 2, Figure 1A), with the number of reports increasing from 329 in 2004 to 1,397 in 2023. This trend may be attributed to factors such as an aging population, increased drug use, and improved AE monitoring (Verhamme et al., 2008). In our study, the proportion of male patients (53.4%) was higher than that of female patients (37.2%), consistent with previous findings (Verhamme et al., 2008; Crisafulli et al., 2022). Statistically, acute UR has an incidence rate of 4.5–6.8 per 1,000 men aged over 70 per year (Crisafulli et al., 2022), while in women, the incidence is about 0.07 per 1,000 (Klarskov et al., 1987). This discrepancy may be linked to men’s higher susceptibility to prostate-related conditions, which increase the risk of UR (Crisafulli et al., 2022). In addition, we also observed that the top six countries in terms of reported cases listed in Table 2 are all developed countries. This may be attributed to their well-established pharmacovigilance systems, higher levels of public and physician awareness, and stricter legal and regulatory requirements.

Our study revealed that the proportion of UR reports related to Antineoplastic Agents and Antidiabetic Drugs showed a significant annual increase from 2004 to 2023 (p-adjust < 0.05), whereas reports related to Bronchodilators exhibited a significant decline. The increase in Antineoplastic Agent-related reports could be due to factors such as increased use, neurotoxicity of the drugs, side effects, and comorbidities in cancer patients (e.g., benign prostatic hyperplasia and diabetes) (Drake et al., 1998; Carbone et al., 2017; Alberti, 2019). The improved drug safety monitoring system and increased patient awareness may also contribute to the higher reporting numbers (Hazell and Shakir, 2006). The increasing proportion of reported UR associated with antidiabetic drugs over the past 20 years can be attributed to several factors. First, the global prevalence of diabetes, particularly the rising burden of type 2 diabetes, has driven a growing demand for antidiabetic medications (Ong et al., 2023). As the incidence of diabetes has increased, new drug classes, including glucagon-like peptide-1 (GLP-1) receptor agonists, sodium-glucose cotransporter 2 (SGLT2) inhibitors, and dipeptidyl peptidase-4 (DPP-4) inhibitors, have been rapidly developed and widely applied (Ahmad et al., 2022). While these drugs offer notable advantages in efficacy, their potential adverse effects, especially those impacting the autonomic nervous system and urinary tract, have not been fully recognized. Additionally, the widespread adoption of personalized treatment strategies has played a significant role. Updates to clinical guidelines, which focus on tailoring treatment plans based on patient characteristics, have led to a more diverse range of medications being prescribed, further increasing their use (Williams et al., 2022). Together, these factors, alongside continuous innovations in diabetes care, help explain the rise in UR reports associated with antidiabetic drugs in recent decades. In contrast, the decrease in Bronchodilator-related UR reports may be related to optimized treatment strategies, improved drug combinations, enhanced patient education, and better drug safety profiles (Rodrigo and Castro-Rodríguez, 2012; Cazzola and Matera, 2014). These findings underscore the importance of monitoring and managing drug-related AEs in clinical practice, especially for high-risk drugs and patient populations.

Certain antispasmodic drugs used for treating overactive bladder, including the anticholinergic agents Fesoterodine (ROR = 91.93) and Solifenacin (ROR = 22.78), and the β3-adrenergic receptor agonist Mirabegron (ROR = 35.46), exhibited high signal strength for UR AEs. Notably, UR is a known common adverse reaction for these drugs. This finding, consistent with their product labels, further validates the safety concerns associated with these drugs in clinical use. Therefore, clinicians should carefully evaluate patient risk factors when prescribing these medications and closely monitor for UR AEs.

Some drugs unexpectedly identified as potentially causing UR—Abiraterone, Fluoxetine, and Empagliflozin—showed positive AE signals in both the FAERS and CVAR databases. This finding is highly significant for drug safety monitoring and risk management, providing a scientific basis for improving drug labeling. Health professionals should exercise increased vigilance when prescribing these medications, particularly to high-risk populations such as older adults or individuals with comorbid conditions. Abiraterone, a selective androgen synthesis inhibitor, reduces androgen synthesis by inhibiting the enzyme cytochrome P450 c17 (CYP17), which is crucial in testosterone production in the adrenal glands, testes, and prostate tumors (de Bono et al., 2011). Beck et al. reported a case of a 74-year-old male developing UR and acute kidney injury with hypokalemia and metabolic alkalosis while on Abiraterone for metastatic prostate cancer. These symptoms resolved upon discontinuation of Abiraterone, suggesting a potential association (Beck et al., 2021), supporting our findings.

Fluoxetine, a selective serotonin reuptake inhibitor (SSRI), is effective and well-tolerated for treating depression and obsessive-compulsive disorder (Bulut et al., 2022). Previous studies have reported UR when Fluoxetine is combined with other antipsychotic or benzodiazepine drugs (Lock et al., 1990; Benazzi, 1996). There are also reports of UR with Fluoxetine monotherapy (Karadag et al., 2015; Bulut et al., 2022). For instance, a 17-year-old female developed UR within the first week of Fluoxetine (20 mg/day) treatment, which worsened to complete inability to urinate. Her symptoms resolved after discontinuing Fluoxetine (Karadag et al., 2015). BULUT also reported a case of chronic UR in a 15-year-old girl on Fluoxetine monotherapy (Bulut et al., 2022). The mechanism by which Fluoxetine causes UR is not fully understood, but several possible explanations exist. First, Fluoxetine may increase the activity of the external urethral sphincter by inhibiting the reuptake of serotonin around Onuf’s nucleus motor neurons (Karadag et al., 2015). Second, blocking spinal 5-HT1a receptors can reduce bladder contractions, thereby promoting the development of UR (Le Poul et al., 2000; Burgard et al., 2003). Clinicians should be aware of the potential for Fluoxetine to cause UR and intervene promptly with timely diagnosis and treatment.

Empagliflozin, a potent selective SGLT2 inhibitor used to treat type 2 diabetes in adults, has a well-documented efficacy and tolerability profile (Frampton, 2018). Its label notes a higher incidence of urinary tract infections (UTIs) in patients, particularly those with a history of chronic or recurrent UTIs, but does not mention UR as a potential AE. Existing studies on Empagliflozin-related UR are scarce. Brock reported a case of asymptomatic UR and emphysematous cystitis in a 62-year-old male with type 2 diabetes on Empagliflozin (Brock et al., 2022). Another drug with a similar mechanism, Dapagliflozin, has been reported to potentially cause UR (Crisafulli et al., 2022). The mechanism behind these drugs inducing UR is unclear but may be linked to their association with UTIs, leading to urethral edema and UR (Serlin et al., 2018). Additionally, diabetes itself can affect bladder nerves, causing bladder dysfunction and potentially leading to UR (Sakakibara et al., 2018). Future studies are needed to clarify whether this potential signal is due to Empagliflozin’s independent effect or a synergistic effect with diabetes.

While Valacyclovir, Clopidogrel, and Amlodipine did not show positive signals for UR in the CVAR validation, this does not rule out their association with the AE. For example, Amlodipine, a calcium channel blocker (CCB), might cause UR by reducing the contractility of smooth muscles, including the bladder detrusor muscle, leading to incomplete bladder emptying (Serlin et al., 2018). Positive signals in the FAERS database suggest potential risks, but these signals may not replicate in validation databases due to sample size, observation time, or other variables. Therefore, clinical observation and further research and monitoring are needed to establish the relationship between these drugs and UR.

Our study found that the median time to onset for drug-related UR was 29 days. Over half of the patients experienced the target AE within the first 30 days of medication use (50.7%), indicating that drug-related UR primarily occurs early in the treatment course. This finding highlights the need for close monitoring of patients during this critical period and underscores the importance of educating patients about the early symptoms of UR to enable prompt intervention. However, it is important to note that UR can still occur over a year after starting the medication.

However, this study has several limitations. First, FAERS and CVAR are based on self-reporting systems, which carry the risks of underreporting, duplicate reporting, and inaccurate reporting. Although we conducted deduplication, the study results may still be biased. Second, there is a lack of overall information on the medication population, making it impossible to calculate the incidence of drug-related UR. Additionally, factors such as patient gender, age, race, comorbidities, and concomitant medications potentially influence the occurrence of AEs, but there are currently no established methods to account for these factors in disproportionality analysis. Furthermore, FAERS does not provide aggregated data for more than five drugs at a time (Giunchi et al., 2023), so our focus was limited to the top 50 drugs reporting UR AEs, a common practice in similar studies (Yu et al., 2021; Fei et al., 2023). Finally, our analysis is primarily hypothesis-generating; thus, the relationship between drugs and UR is correlational rather than causal. Potential safety signals need further evaluation through pharmacoepidemiological studies.

## 5 Conclusion

Our analysis of FAERS data reveals a consistent upward trend in reports of drug-induced UR over the past 2 decades. Notably, there has been a significant increase in UR reports associated with antineoplastic and antidiabetic drugs, while those linked to bronchodilators have decreased. The CVAR analysis has validated the newly identified signals for Abiraterone, Fluoxetine, and Empagliflozin. These findings are vital for healthcare providers, researchers, and regulatory authorities, highlighting the critical need for continuous monitoring and reassessment of drug safety to safeguard patient health. Furthermore, there is a pressing need for comprehensive clinical and pharmacoepidemiological studies to deepen our understanding of the mechanisms driving drug-induced UR.

## Data Availability

The original contributions presented in the study are included in the article/Supplementary Material, further inquiries can be directed to the corresponding author.
