# Supplementary material for: Drug-induced urinary retention: a real-world pharmacovigilance study using FDA and Canada vigilance databases
Source: Front Pharmacol. 2025 Jan 6;15:1466875. doi: 10.3389/fphar.2024.1466875 (PMC11744018; doi:10.3389/fphar.2024.1466875)
Supplement: Supplementary file 2 [file Table1.docx]

**Supplementary Table S1.** Global reporting of drug-related UR events by country. UR, Urinary retention.

| **Reported Countries** | Number of Cases | Percentage |
| --- | --- | --- |
| United States | 5348 | 33.05% |
| Japan | 1712 | 10.58% |
| France | 1431 | 8.84% |
| United Kingdom | 1344 | 8.31% |
| Germany | 976 | 6.03% |
| Canada | 756 | 4.67% |
| Italy | 370 | 2.29% |
| China | 332 | 2.05% |
| Spain | 328 | 2.03% |
| Netherlands | 288 | 1.78% |
| Sweden | 267 | 1.65% |
| Australia | 234 | 1.45% |
| Poland | 170 | 1.05% |
| Brazil | 125 | 0.77% |
| Switzerland | 114 | 0.70% |
| Austria | 110 | 0.68% |
| Denmark | 105 | 0.65% |
| Portugal | 100 | 0.62% |
| Turkey | 86 | 0.53% |
| Israel | 81 | 0.50% |
| Belgium | 79 | 0.49% |
| South Korea | 75 | 0.46% |
| India | 72 | 0.44% |
| Czech Republic | 71 | 0.44% |
| Ireland | 58 | 0.36% |
| Norway | 56 | 0.35% |
| Russia | 56 | 0.35% |
| Croatia | 49 | 0.30% |
| Greece | 44 | 0.27% |
| Colombia | 39 | 0.24% |
| Finland | 39 | 0.24% |
| Romania | 32 | 0.20% |
| Slovenia | 29 | 0.18% |
| South Africa | 29 | 0.18% |
| Hungary | 20 | 0.12% |
| Slovakia | 20 | 0.12% |
| Malaysia | 17 | 0.11% |
| Argentina | 16 | 0.10% |
| Egypt | 15 | 0.09% |
| New Zealand | 14 | 0.09% |
| Philippines | 14 | 0.09% |
| Mexico | 13 | 0.08% |
| Saudi Arabia | 13 | 0.08% |
| Singapore | 13 | 0.08% |
| Iran | 12 | 0.07% |
| Congo | 8 | 0.05% |
| Indonesia | 8 | 0.05% |
| Lithuania | 8 | 0.05% |
| Serbia | 8 | 0.05% |
| Thailand | 8 | 0.05% |
| Vietnam | 7 | 0.04% |
| Chile | 6 | 0.04% |
| Jordan | 6 | 0.04% |
| Latvia | 6 | 0.04% |
| Lebanon | 6 | 0.04% |
| Nigeria | 6 | 0.04% |
| Bulgaria | 5 | 0.03% |
| Costa Rica | 5 | 0.03% |
| Iceland | 5 | 0.03% |
| Puerto Rico | 5 | 0.03% |
| Sri Lanka | 5 | 0.03% |
| Tunisia | 5 | 0.03% |
| Ukraine | 5 | 0.03% |
| Algeria | 4 | 0.02% |
| Ghana | 4 | 0.02% |
| Togo | 4 | 0.02% |
| Belarus | 3 | 0.02% |
| Panama | 3 | 0.02% |
| Bangladesh | 2 | 0.01% |
| Georgia | 2 | 0.01% |
| Kazakhstan | 2 | 0.01% |
| Kenya | 2 | 0.01% |
| Kuwait | 2 | 0.01% |
| Montenegro | 2 | 0.01% |
| Nicaragua | 2 | 0.01% |
| Antigua and Barbuda | 1 | 0.01% |
| Botswana | 1 | 0.01% |
| Cameroon | 1 | 0.01% |
| Cyprus | 1 | 0.01% |
| Dominican Republic | 1 | 0.01% |
| Ecuador | 1 | 0.01% |
| Estonia | 1 | 0.01% |
| Luxembourg | 1 | 0.01% |
| Malta | 1 | 0.01% |
| Moldova | 1 | 0.01% |
| Morocco | 1 | 0.01% |
| Pakistan | 1 | 0.01% |
| Peru | 1 | 0.01% |
| Qatar | 1 | 0.01% |
| United Arab Emirates | 1 | 0.01% |
| Uruguay | 1 | 0.01% |
| Venezuela | 1 | 0.01% |
| Yemen | 1 | 0.01% |
| Unknown | 849 | 5.25% |
